# Noncoding RNAs and cancer

**DOI:** 10.1186/1758-907X-2-6

**Published:** 2011-09-29

**Authors:** Ohad Yogev, Dimitris Lagos

**Affiliations:** 1 Cancer Research UK Viral Oncology Group, UCL Cancer Institute, University College London, Paul O'Gorman Building, 72 Huntley Street, London, WC1E 6BT, UK; 2 Centre for Immunology and Infection, Department of Biology and Hull York Medical School, University of York, Wentworth Way, Heslington, York, YO10 5DD, UK

**Keywords:** noncoding RNA, cancer, microRNA

## Abstract

The study of miRNAs and other noncoding RNAs has revolutionised our understanding of gene expression regulation during cancer development and progression, creating one of the fastest-growing research fields in cancer with realistic therapeutic potential. The 2011 Non-coding RNAs and Cancer Symposium hosted by the University College London Cancer Institute focused on the function and regulation of noncoding RNAs during oncogenesis.

## Introduction

Understanding the mechanisms that regulate gene expression during cancer development is of paramount importance for the development of effective therapeutic regimens. The discovery of miRNAs, a class of noncoding RNA genes with a role in gene silencing [1-3], caused a dramatic increase in research activity aimed at unravelling the role of noncoding RNAs in cancer. It has now become apparent that it is necessary to study the function of miRNAs and other noncoding RNAs, which account for almost 40% of the human genome [4], and integrate these findings with our understanding of the functions of protein-coding genes, which compose almost 2% of the human genome, in cancer. During the 2011 Non-coding RNAs and Cancer Symposium in London, some fascinating aspects of the role of noncoding RNAs in cancer were discussed.

### miRNAs as oncogenes and tumour suppressors

miRNAs are a class of small noncoding RNAs, approximately 22 nucleotides long, that are involved in posttranscriptional gene regulation. They arise from intergenic or intragenic genomic regions and are transcribed as long primary transcripts. These primary transcripts undergo two processing steps that produce the mature form of the miRNA. Once processed, miRNAs are loaded into the RNA-induced silencing complex (RISC), directing it to target mRNAs and causing posttranscriptional repression [5,6]. The discovery of miRNAs has led to profound changes in the understanding of eukaryotic gene-regulatory networks. Functional studies indicate that miRNAs participate in the regulation of almost every cellular process examined, and changes in their expression characterise several human diseases, including cancer. miRNAs constitute about 3% to 5% of predicted genes in the human genome, and about one-fourth of protein-coding genes are estimated to be regulated by them [7]. A growing amount of evidence proves that miRNAs can work as oncogenes by activating the malignant potential of cells or, conversely, as tumour suppressor genes by blocking this potential [5,8]. However, since specific miRNAs can regulate different targets in different tissues, one cannot describe them as tumour suppressors or oncogenes before specifying the tissue of their action [8].

One of the first lines of evidence that miRNAs can act as oncogenes or tumour suppressors came from the discovery of the role of miR-16-1 and miR-15a in chronic lymphocytic leukaemia (CLL), as presented by Carlo M Croce (Human Cancer Genetics Program, The Ohio State University Medical Center, Columbus, OH, USA). During attempts to clone a tumour suppressor gene at 13q14, a chromosomal region that is frequently lost in CLL, the CLL suppressor gene was found to be located in a small genomic region in which there are no protein-coding genes. However, two miRNA genes, *miR-15a *and *miR-16-1*, are located within this region. This indicates that *miR-15a *and *miR-16-1 *can function as tumour suppressors and that their loss is associated with the development of the indolent form of CLL [9]. Following this discovery, Croce and colleagues mapped the chromosomal locations of other known miRNAs, and surprisingly, they found that many miRNA genes are located within regions that are frequently altered in many types of human cancer [10]. In the case of *miR-16-1 *and *miR-15a *in CLL, the two miRNAs act as tumour suppressors by suppressing expression of *BCL2*, an oncogene that inhibits apoptosis and whose overexpression appears to be a crucial event during the initiation of most forms of the disease [11,12].

On the other hand, miR-155 is overexpressed in aggressive CLL and acts as an oncogene in CLL [13]. It has been suggested that miR-155 enhances the mutation rate of CLL by targeting genes involved in DNA repair and cell cycle regulation [14,15]. Moreover, transgenic mice with targeted overexpression of miR-155 in B cells developed a polyclonal expansion of large pre-B cells followed by leukaemia or high-grade lymphoma, demonstrating that a miRNA can contribute directly to the pathogenesis of malignancy [16]. These results indicate that the dysregulation of a single miRNA can lead to the development of a malignant tumour. Following the above-described seminal discoveries, several miRNAs have been shown to act as tumour suppressors or oncogenes [8].

### miRNAs in tissue development and degeneration

miRNAs have been shown to play a central role in cancer angiogenesis [17,18]. The study of miRNAs during normal vascular development *in vivo *has provided useful insight into miRNA function in pathological angiogenesis. To this purpose, the use of zebrafish provides an ideal model for uncovering the contribution of individual miRNAs in development. Using this approach, Antonio Giraldez and colleagues (Department of Genetics, Yale University, New Haven, CT, USA) described the identification of 245 mRNAs that are posttranscriptionally regulated by muscle miRNAs in zebrafish. Two muscle-specific miRNAs, miR-1 and miR-133, appear to instruct embryonic muscle gene expression and to downregulate specific targets in muscle. They also identified a set of targets with relatively low expression in muscle tissue whose downregulation is miRNA-independent. This led them to suggest that there are two modes of gene regulation in muscle cells: the first is governed by miRNAs, and the second is primarily regulated at the transcriptional level with miRNAs acting only to fine-tune expression level. Furthermore, they found a number of actin-related and actin-binding proteins among the miR-1 and miR-133 targets, suggesting that these miRNAs regulate sarcomeric actin organization [19]. Intriguingly, the group also found that some muscle-specific miRNAs may also play a role in angiogenesis during zebrafish development.

Interestingly, the involvement of miRNAs in cell proliferation and function is also demonstrated through studies of other diseases, such as neurodegenerative conditions. In this respect, Eran Hornstein (The Weizmann Institute of Science, Rehovot, Israel) presented a model for spinal motor neuron (SMN) disease that is based on loss of *Dicer1 *function. It is already well established that posttranscriptional gene regulation plays a crucial role in the development and function of neurons, and alterations in miRNA function have been found to contribute to neuronal disease susceptibility. In addition, several RNA-binding proteins involved in the miRNA biogenesis pathway were also found to be mutated in neuronal diseases such as amyotrophic lateral sclerosis (ALS). Deep sequencing was used to investigate the neuronal miRNA milieu, which was found to be dominated by four miRNAs: both arms of miR-9, Hoxmir and let-7. To explore the involvement of miRNAs in the pathogenesis of motor neuron (MN) disease, an MN *Dicer*-mutant mouse was created. These mice have denervation muscular atrophy, which suggests loss of SMNs, and exhibit a significant decrease in MN axon numbers. It has previously been shown that the coordinated expression levels of the neurofilament subunit proteins are disturbed in human ALS, which can be caused by upregulation of the heavy subunit. Hornstein's group [20] suggested that miR-9 coordinates the expression of the neurofilament subunits by regulating the expression of the heavy subunit. This hypothesis was reinforced when they found that miR-9 is also specifically downregulated in other models of MN disease.

### miRNAs and epigenetic switches

Kevin Struhl (Department of Biological Chemistry and Molecular Pharmacology, Harvard Medical School, Boston, MA, USA) described a link between miRNAs and epigenetic changes that occur in an inducible model of cellular transformation. In this model, nontransformed mammary epithelial cell lines containing oestrogen receptor and Src are treated with tamoxifen. This treatment rapidly induces Src, and morphological transformation is observed within 36 hours. Src activation triggers an inflammatory response that results in an epigenetic switch between nontransformed and transformed cells. The epigenetic switch is mediated by a positive feedback loop involving NF-κB, Lin28b, let-7 and IL-6 [21]. This regulatory circuit is not exclusive to this model and operates in other cancer cell lines, and its transcriptional signature is found in patient cancer tissues. They used this model to look for miRNAs, the expression of which is changed during the course of transformation. Intriguingly, they found two miRNAs, miR-21 and miR-181b-1, that not only are overexpressed during transformation, but transient expression of either of them is sufficient to induce a stable transformed state. This suggests that these miRNAs are part of the regulatory circuit, and indeed they found that their expression is regulated by IL-6 and that both miR-21 and miR-181b-1 can activate NF-κB by targeting and inhibiting the tumour suppressors PTEN and CYLD [22].

The Croce group also found that miRNAs regulate epigenetic changes. An example is the miR-29 family, which is downregulated in acute leukaemias and targets (directly and indirectly) several DNA methyltransferases. Introduction of the miR-29 family into lung cancer cell lines caused demethylation of the CpG islands in the promoter regions of tumour suppressor genes, which allowed their reactivation and resulted in loss of tumourigenicity [23,24].

### Making sense of the mess

miRNAs bind their target mRNAs through base pairing, which occurs primarily between positions 2 and 8 of the mature miRNA and sequences in the 3'UTR of the target mRNA [6]. Because of the nature of this molecular targeting mechanism, one of the biggest challenges in the field of miRNAs is distinguishing biologically relevant miRNA-mRNA interactions. Until recently, identification of miRNA target sites predominantly relied on computational methods that are limited in their ability to predict specific and physiologically relevant targets [25]. Lately several studies have addressed this problem by utilising immunoprecipitation of miRNA effector complexes consisting of one of the Argonaute proteins (the central protein component of RISC) cross-linked with associated miRNAs and mRNAs. This cross-linking and immunoprecipitation (CLIP), coupled with deep sequencing, provides transcriptome-wide coverage as well as high resolution. However, partly because it is so vast, the data that have so far been generated in CLIP experiments have not yet been put in a form that enables fast and interactive exploration of binding sites. Mihaela Zavolan (The Center for Molecular Life Sciences, University of Basel, Basel, Switzerland) presented a new database named CLIPZ that was developed for this purpose. This is a database of binding sites that were constructed based on CLIP data for various RNA-binding proteins (RBPs), which are known to regulate mRNA splicing, stability and/or translation rate [26].

### The increasing arsenals of noncoding RNAs

Although miRNAs are the most frequently studied RNAs, they comprise only a small portion of the cellular noncoding RNA. The development of deep sequencing technologies and the improved analysis tools have allowed the identification of new groups of small noncoding RNA. In his talk, Gyorgy Hutvagner (Wellcome Trust Centre, Dundee, UK) described how, by a combination of *in silico *analysis with *in vivo *and *in vitro *experiments, his research group was able to identify a new group of small RNAs, which are generated after processing of mature or precursor transfer RNAs (tRNAs). This process gives rise to two types of tRNA-derived RNA fragments (tRFs), 5'tRFs and 3'tRFs, produced from the 5' and 3' ends of the tRNA, respectively. Moreover, the formation of these tRFs is dependent on Dicer activity [27].

Richard Jenner (Department of Infection and Immunity, University College London, London, UK) presented another new class of short RNAs that are transcribed from the 5' end of polycomb target genes. Polycomb group proteins are essential for embryogenesis and for maintaining embryonic stem (ES) cells' pluripotency and differentiated states. PRC2 is a polycomb repressive complex that catalyses the trimethylation of lysine 27 of histone H3, forming a binding site for PRC1. This enables the repression of hundreds of developmental regulators in ES cells that would otherwise induce cell differentiation. Although repressed, PCR2 target genes are associated with histone H3K4me3, a marker of transcription initiation. In addition, it has been shown that PRC2 can interact with long noncoding RNA transcripts such as RepA or HOTAIR. In the course of the Jenner group's work, they identified a new class of short RNAs, 50 to 200 nucleotides long that are transcribed from the 5' end of polycomb target genes. These short RNAs interact with PRC2 through a stem-loop structure and cause gene repression in *cis*. During cell differentiation, these RNAs are depleted from the polycomb targets. This new model can explain why polycomb target genes are associated with transcriptional activation markers and provides a potential new role for small RNAs in the interaction of PRC2 with its target genes [28].

### RNA-protein interactions in cancer

Posttranslational regulation through interaction between mRNAs and RBPs occurs in a small RNA-dependent or -independent manner. Examples of small RNA-dependent interactions include the above-mentioned gene expression suppression by RISC or PRC. Martin Turner (Babraham Institute, Cambridge, UK) presented findings highlighting the function of RBPs in T-lymphocytes and leukaemia. TIS11b and TIS11d are RBPs that interact with AU-rich elements in the 3'UTR of mRNA, which leads to mRNA degradation and translational repression. Turner's research group has shown that mice lacking these proteins during thymopoiesis develop T-cell acute lymphoblastic leukaemia (T-ALL). They found that these RBPs bind to the 3'UTR of the transcription factor Notch1 and by doing so, suppress its expression. The absence of these two RBPs leads to higher expression of Notch1, which can cause perturbation and higher metabolic activity. Finally, developing T-ALL in their model was shown to be Notch1-dependent, suggesting that TIS11b and TIS11d can act as tumour suppressors. Indeed, these proteins are dysregulated in several different types of cancer. These results demonstrate the critical role of RBPs in thymocyte development and in the prevention of transformation [29].

### miRNAs as therapeutics

miRNAs play an important role in many different disorders, particularly in cancer, where they have been shown to act as both tumour suppressors and oncogenes. They have also been shown to function in viral defence and can prevent viral infection. Sakari Kauppinen (Santaris Pharma A/S, Hørsholm, Denmark) presented a new approach that enables miRNA antagonism using tiny, locked nucleic acids (tiny LNAs). These are fully modified phosphorothionate oligonucleotides, which are complementary to the miRNA seed region. Since miRNA families share the same seed sequence, the great advantage of tiny LNAs is that a single molecule is able to repress an entire miRNA family, as shown for the let-7 family. More importantly, they demonstrated that systematically delivered, unconjugated tiny LNAs showed uptake into many normal tissues and into breast tumours in mice, which coincided with long-term miRNA silencing. Using a specific LNA, they were able to inhibit *miR-21*, a known miRNA oncogene, both *in vitro *and *in vivo*, and prevent its oncogenic effect [30].

LNA-mediated miRNA antagonism is also used for prevention of viral infection in the most advanced clinical trial targeting a miRNA http://www.santaris.com/product-pipeline. miR-122 binds to two closely spaced target sites in the 5' noncoding region of the hepatitis C virus (HCV) genome, resulting in upregulation of viral RNA levels. Interaction of miR-122 with the HCV genome is essential for accumulation of viral RNA in cultured liver cells. Treatment of chronically infected chimpanzees with a LNA complementary to miR-122 leads to long-lasting suppression of HCV viraemia with no evidence of viral resistance [31]. Following these studies in preclinical models, and after successful completion of phase I clinical trials of these compounds in humans, the phase II trial is now ongoing for treatment of HCV.

### A noncoding RNA world

Individual miRNAs have multiple targets, which in principle can compete against each other for binding to the miRNA. Therefore, one can assume that an independent change in the expression of one RNA in this network will affect the levels of all the rest. This network can include not only mRNAs but also noncoding RNAs such as pseudogenes. This theory expands on the central dogma, since it means that a gene does not have to be translated to have a function. This hypothesis was first suggested by Pier-Paolo Pandolfi and was termed 'the ceRNA hypothesis'. Dr Pandolfi (Beth Israel Deaconess Medical Center, Harvard Medical School, Boston, MA, USA) described the function of gene and pseudogene mRNAs in tumour biology as a model for the protein coding-independent role of RNAs. In this work, they tested the relationship between *PTEN *and its pseudogene *PTEN1*. *PTEN *is downregulated in 70% of human cancers, and there are several indications that it functions as a haploinsufficient tumour suppressor gene [32]. *PTEN *expression is downregulated by several different miRNAs, and it was demonstrated that posttranscriptional regulation has a pivotal role in determining *PTEN *abundance in cancer cells. The pseudogene *PTEN1 *is conserved, and its 3'UTR includes miRNA recognition elements shared with the *PTEN *3'UTR. In their work, Pandolfi and colleagues found that the pseudogene *PTEN1 *is biologically active, as it regulates *PTEN *expression by sequestering shared miRNAs, preventing them from binding to the 3'UTR of *PTEN*. They found that expression of the *PTEN1 *3'UTR alone was sufficient to cause overexpression of *PTEN *and prevent tumourigenesis. They also found that the *PTEN1 *locus is selectively lost in human cancers. These results suggest that *PTEN1*, despite its not coding for a protein, can act as a tumour suppressor gene. These findings point towards a new layer of complexity in the field of the noncoding RNAs and their role in posttranscriptional regulation. According to this model, when identifying the miRNA recognition elements in a specific gene, it will also be necessary to look for these elements in other genes. This will allow the elucidation of the full network of noncoding RNAs that regulate expression of a particular gene [32].

## Conclusions

The 2011 Non-coding RNAs and Cancer Symposium highlighted the role of miRNAs and other noncoding RNAs as crucial molecular switches in cancer. During the meeting, it emerged that there are still exciting challenges in understanding the function and regulation of the various noncoding RNA classes in cancer. These challenges include the accurate and unbiased identification of miRNA targets, the elucidation of the role of novel classes of noncoding RNAs in cancer (such as tRFs and polycomb-associated RNAs) and the in-depth investigation of direct interactions between noncoding RNAs and their relevance to cancer biology. However, it also became apparent that the use of noncoding RNA-based therapeutics and diagnostics in cancer medicine is fast approaching.

## Abbreviations

IL: interleukin; miRNA: microRNA; NF-κB: nuclear factor κB.

## Competing interests

The authors declare that they have no competing interests.

## Authors' contributions

DL and OY wrote this meeting report.
